# Intravascular Large B-cell Lymphoma Presenting as Hemophagocytic Lymphohistiocytosis and Pyrexia of Unknown Origin: A Diagnostic Dilemma

**DOI:** 10.7759/cureus.97494

**Published:** 2025-11-22

**Authors:** Mohammed Haneef Aloodigothi, Anand Anilkumar, Ameera Kunhayamed

**Affiliations:** 1 Internal Medicine, Aster MIMS Kannur, Kannur, IND

**Keywords:** bone marrow biopsy, hemophagocytic lymphohistiocytosis (hlh), intravascular large b-cell lymphoma, positron emission tomography-computed tomography, pyrexia of unknown origin (puo)

## Abstract

Intravascular large B-cell lymphoma (IVBCL) is a rare, aggressive subtype of diffuse large B-cell lymphoma distinguished by the proliferation of malignant lymphoid cells within the lumina of small vessels. Its unusual growth pattern results in absent lymphadenopathy and nonspecific imaging findings, often leading to delayed diagnosis. We report the case of a 71-year-old man who presented with prolonged fever, hepatosplenomegaly, anemia, leukopenia, thrombocytopenia, and markedly elevated ferritin. He was diagnosed with secondary hemophagocytic lymphohistiocytosis (HLH) and initially improved with HLH-directed immunosuppressive therapy but soon deteriorated again. PET-CT showed only diffuse hepatic and splenic uptake without discrete lesions. A bone marrow biopsy revealed IVBCL. This case highlights the diagnostic challenges of IVBCL, particularly its overlap with HLH, the limitations of PET-CT, and the crucial role of bone marrow biopsy. Early detection and initiation of therapy remain essential for improving outcomes.

## Introduction

Pyrexia of unknown origin (PUO) was first defined in 1961, and although diagnostic tools have advanced considerably, a significant proportion of patients remain undiagnosed after thorough evaluation [1]. Among the causes of PUO, both hemophagocytic lymphohistiocytosis (HLH) and intravascular large B-cell lymphoma (IVBCL) warrant particular attention because of their nonspecific presentations and substantial clinical overlap.

HLH is a fatal hyperinflammatory syndrome characterized by uncontrolled immune activation leading to fever, cytopenias, splenomegaly, hyperferritinemia, and multiorgan dysfunction [2]. Among malignancies, hematologic neoplasms are particularly important in adults. When HLH arises secondary to an occult malignancy, diagnosis becomes especially challenging due to rapid clinical deterioration and the lack of early, disease-specific clues.

IVBCL is a rare, aggressive extranodal subtype of diffuse large B-cell lymphoma, with an incidence of less than 0.5 per million per year [3,4]. It is characterized by proliferation of malignant B-cells confined to small vessels, typically without lymphadenopathy or tumor masses, making conventional imaging and clinical evaluation unreliable [3]. IVBCL often presents with nonspecific symptoms such as persistent fever, PUO, cytopenias, neurological findings, or skin involvement, and its protean manifestations contribute to frequent delays or missed diagnoses [4]. Two variants are recognized: the Western form, characterized by predominant involvement of the skin and central nervous system, and the Asian form, which more often presents with systemic symptoms, cytopenias, hepatosplenomegaly, and HLH-like manifestations [3,4].

The association between IVBCL and secondary HLH is well documented and reflects the intense cytokine activation driven by intravascular lymphoma cells [2]. When IVBCL presents primarily with HLH, the underlying malignancy may remain occult unless a bone marrow biopsy is pursued early, often making PUO with HLH a critical diagnostic intersection.

We present the case of an elderly man with PUO and HLH in whom PET-CT was nondiagnostic and bone marrow biopsy ultimately revealed IVBCL, underscoring the diagnostic complexity of this entity and the importance of maintaining early suspicion for IVBCL-associated HLH.

## Case presentation

A 71-year-old man, with nearly a decade of poorly controlled type 2 diabetes and a history of regular alcohol use, presented with three weeks of persistent fever, profound fatigue, anorexia, and intermittent episodes of disorientation.

On admission, he was febrile and visibly jaundiced. Examination revealed pedal edema, hepatosplenomegaly, gynecomastia, and a flapping tremor. Notably, there was no palpable lymphadenopathy or abdominal mass.

Initial laboratory investigations revealed mild thrombocytopenia and impaired liver function (Table 1). The tumor marker CA 19-9 was elevated at 229 U/mL, likely reflecting hepatic dysfunction and cholestasis rather than malignancy [5]. Serum sodium was reduced to 121 mmol/L, and inflammatory markers were elevated (CRP 58 mg/L, ESR 23 mm/hr). Screening for infectious causes, including a tropical fever panel, blood and urine cultures, and viral serologies (HBsAg, HCV, HAV, HIV, EBV, CMV, and Parvovirus B19), was negative.

**Table 1:** Laboratory findings on admission

Parameter	Value	Reference range
Hemoglobin	13.9 g/dL	13-16.5 g/dL
WBC	4,120/cmm	4-10k/cmm
Platelets	108k	150-410k
Sodium	121 mmol/L	135-145 mmol/L
Total/direct bilirubin	2.4/2.0 mg/dL	≤1.2/0.2 mg/dL
Aspartate aminotransferase	123 U/L	≤40 U/L
Alanine transaminase	73 U/L	≤41 U/L
Albumin	2.7 g/dL	3.9-4.9 g/dL
Alkaline phosphatase	779 U/L	40-129 U/L
CRP	58 mg/L	<10 mg/L
Erythrocyte sedimentation rate	23 mm/hr	0-15 mm/hr
CA 19-9	229 U/mL	<37 U/mL

**Figure 1:**
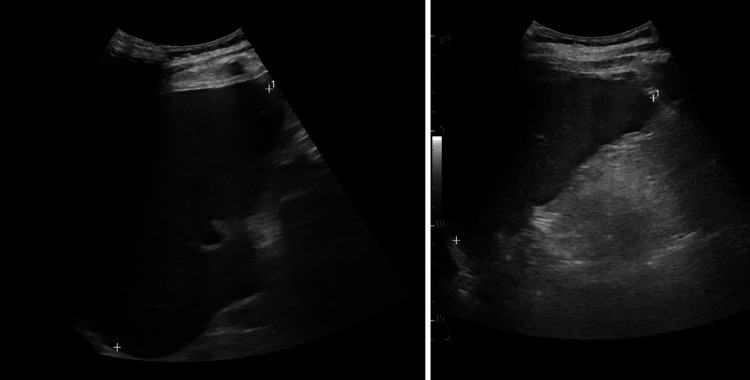
Abdominal ultrasound showing hepatomegaly on the left and splenomegaly on the right

**Figure 2:**
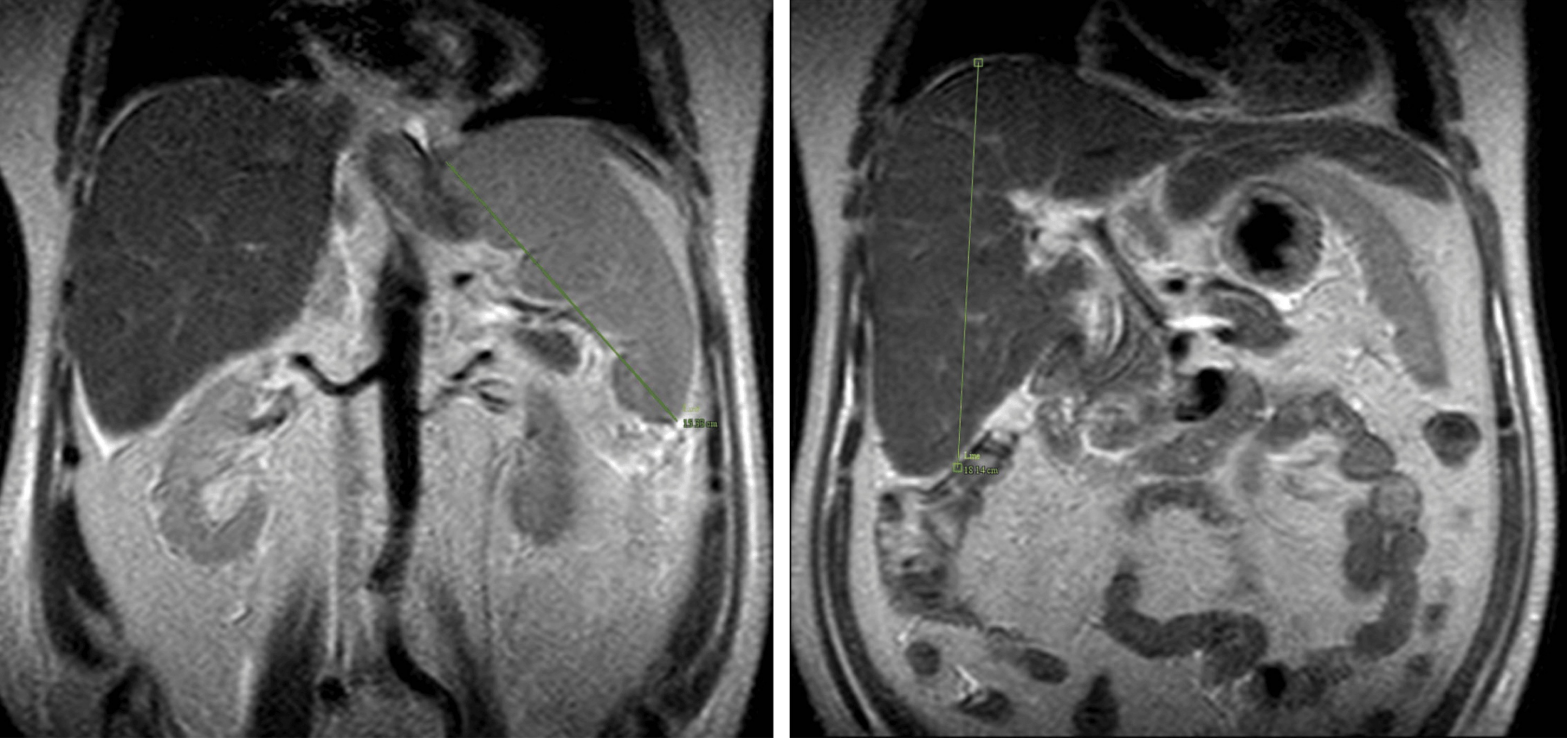
MRI of the abdomen (plain image) MRI of the abdomen (plain image) demonstrating hepatomegaly on the right, with a liver span of 18.14 cm, and splenomegaly on the left, with a splenic span of 15.33 cm.

Ultrasound and MRCP performed towards the end of the first week confirmed hepatosplenomegaly (Figure 1, Figure 2). Despite supportive management, the patient remained febrile and progressed to hepatic encephalopathy, with an ammonia level of 85 µmol/L. His blood parameters began deteriorating more noticeably during the second week, while ferritin and triglycerides rose markedly (Table 2). These findings fulfilled the HLH-2004 diagnostic criteria for secondary HLH [6].

**Table 2:** Laboratory findings during suspected HLH HLH, hemophagocytic lymphohistiocytosis

Parameter	Value	Reference range
Hemoglobin	8.5 g/dL	13-16.5 g/dL
WBC	2,280/cmm	4-10k/cmm
Platelets	103k	150-410k
Ferritin	15,916 ng/mL	30-400 ng/mL
Triglycerides	551 mg/dL	<150 mg/dL
Fibrinogen	470 mg/dL	200-400 mg/dL

**Figure 3:**
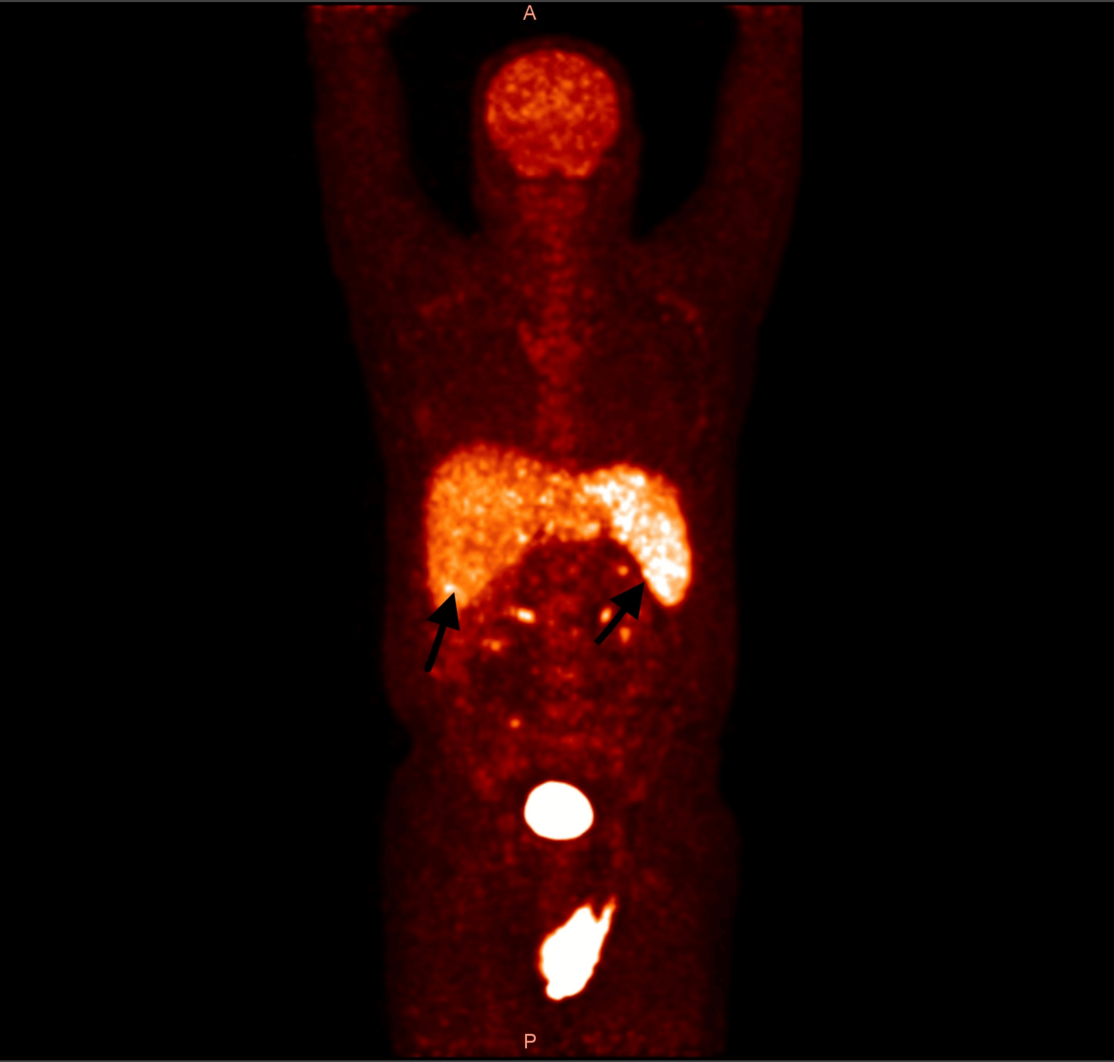
Whole-body PET-CT (coronal view) Diffuse FDG uptake is noted in the enlarged liver and spleen, without evidence of focal lesions or lymphadenopathy. FDG, fluorodeoxyglucose

**Figure 4:**
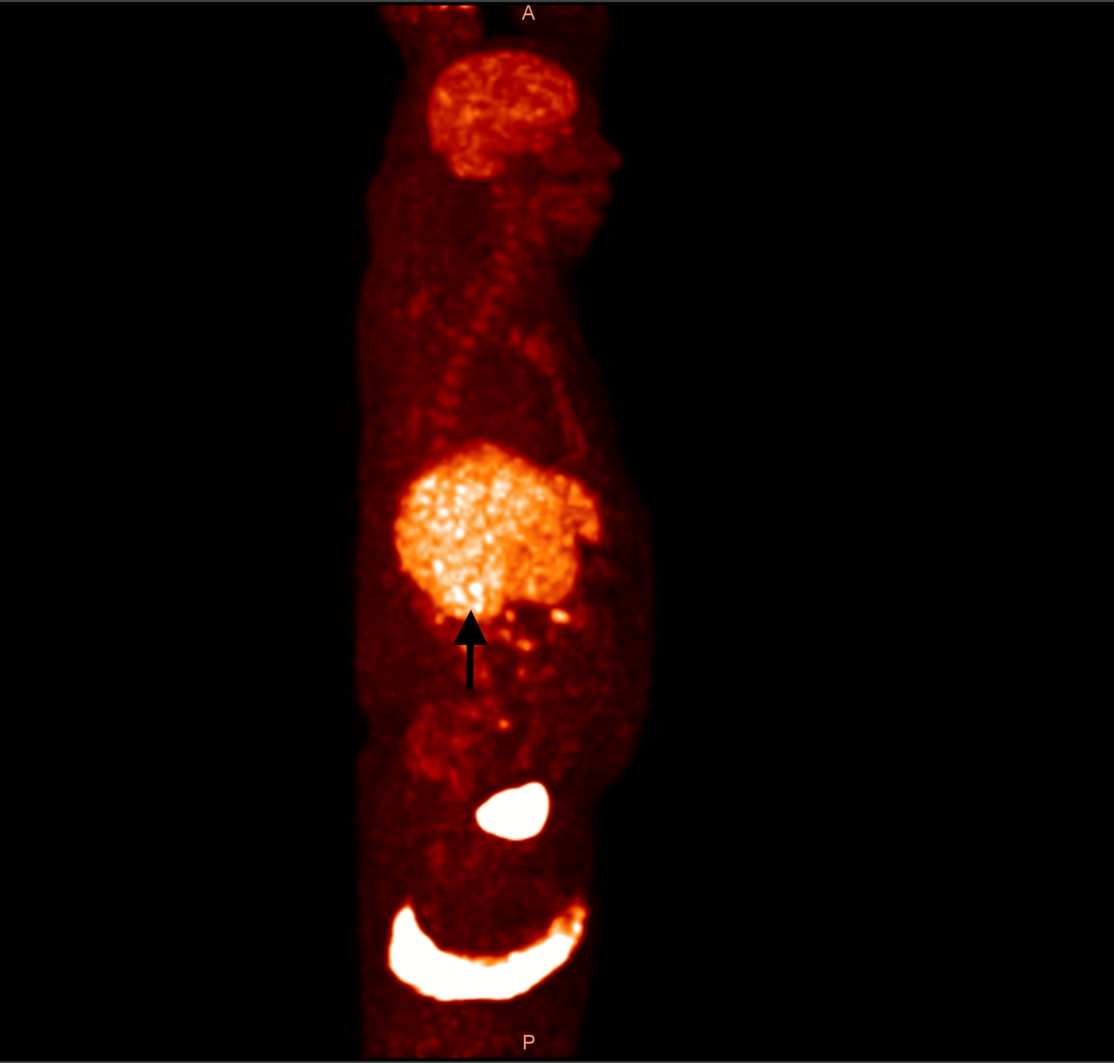
Whole-body PET-CT (sagittal view) Sagittal PET-CT image showing diffuse hepatic and splenic FDG uptake, consistent with inflammatory changes and absence of discrete masses. FDG, fluorodeoxyglucose

A whole-body PET-CT scan, performed around the third week of illness, revealed diffuse fluorodeoxyglucose uptake in the spleen and liver but no focal lesions or lymphadenopathy (Figure 3, Figure 4). With no identifiable infectious, autoimmune, or malignant cause, he was started on an HLH-directed regimen. His fever subsided, and his blood counts improved briefly, but within a week, his cytopenias worsened again (Table 3).

**Table 3:** Laboratory findings after HLH treatment HLH, hemophagocytic lymphohistiocytosis

Parameter	Value	Reference range
Hemoglobin	6.0 g/dL	13-16.5 g/dL
WBC	2,100/cmm	4-10k/cmm
Platelets	130k	150-410k

Given the unexplained course, a bone marrow biopsy was performed almost a month after the initial presentation. It demonstrated a hypercellular marrow with reduced erythroid precursors and interstitial infiltration by atypical large B-cells confined to small vessels. A diagnosis of IVBCL was confirmed on immunohistochemistry, as it confirmed B-cell lineage (Table 4). He was then referred to oncology for initiation of systemic chemotherapy.

**Table 4:** Summary of key diagnostic investigations and findings FDG, fluorodeoxyglucose; HLH, hemophagocytic lymphohistiocytosis; MRCP, magnetic resonance cholangiopancreatography

Investigation	Findings
Ultrasound/MRCP	Hepatosplenomegaly confirmed
PET-CT	Diffuse hepatic and splenic FDG uptake; no focal lesions
Peripheral smear	Cytopenias present; no blasts or abnormal lymphoid cells
Immunohistochemistry	Immunohistochemistry showed that the atypical large lymphoid cells were strongly CD20-positive with dim CD5 expression, confirming B-cell lineage. The neoplastic cells were restricted to vascular lumina, consistent with an intravascular pattern. They were negative for CD34, CD138, MUM1, CD10, and Cyclin D1, effectively excluding plasma cell neoplasms and other B-cell lymphoma subtypes. Non-neoplastic elements included scattered CD3-positive reactive T cells and CD138-positive plasma cells. E-cadherin highlighted scattered proerythroblasts within the marrow.
Bone marrow biopsy	The bone marrow was mildly hypercellular for age, with a diffuse interstitial infiltrate of atypical mononuclear cells. In view of the clinical history of HLH, the absence of lymphadenopathy, and the accompanying immunohistochemical profile demonstrating intravascular B-cell predominance.

## Discussion

This report reflects the challenges that clinicians face in recognizing IVBCL. The patient presented with a fever of unknown origin, hepatosplenomegaly, progressive cytopenias, and a striking rise in ferritin levels. These features met the criteria for HLH as outlined in the HLH-2004 guidelines [6]. HLH is a hyperinflammatory syndrome that can appear in the context of infections, autoimmune disease, or malignancy, and when it occurs as a paraneoplastic manifestation of IVBCL, it often obscures the underlying diagnosis [2].

One potential confounder in this case was the elevation of serum CA 19-9, which initially suggested a gastrointestinal or hepatobiliary malignancy. However, benign hepatic conditions are well documented to cause significant CA 19-9 elevation, particularly in the setting of cholestasis and bile duct inflammation. In fact, hepatic dysfunction accounts for nearly one-third of nonmalignant CA 19-9 elevations [5]. In our patient, with clear evidence of hepatic inflammation and cholestatic dysfunction, the rise in CA 19-9 was most consistent with benign hepatic injury rather than an occult solid tumor.

Imaging did little to clarify the picture. PET-CT has become a cornerstone in the evaluation of lymphoma, but it is not always reliable in IVBCL [7]. Because the malignant cells are confined to the lumina of small vessels, they rarely form discrete masses or enlarged nodes [4]. The result is often a scan that shows either diffuse uptake or no abnormalities at all. Several case reports and series have highlighted these pitfalls, with patients exhibiting only nonspecific uptake or completely normal imaging despite widespread disease [8,9]. Our patient’s PET-CT, which demonstrated only diffuse splenic and hepatic activity, was a typical example of this limitation.

The concept of distinct clinical variants helps explain why IVBCL can present in such diverse ways. The Asian variant is strongly linked with fever, HLH, hepatosplenomegaly, and bone marrow involvement [3,4]. By contrast, the Western form more commonly affects the central nervous system and skin [3,4,10]. In Asian variant cases, such as ours, the presentation often mimics infection-related HLH, which can easily lead clinicians down the wrong path and delay recognition of the lymphoma [2].

Ultimately, it was the bone marrow biopsy that provided the answer. Even when imaging is unrevealing, marrow examination can show the intravascular growth pattern that defines this disease. Earlier reports highlighted this as a critical diagnostic route [11], and more recent descriptions have confirmed that marrow involvement can sometimes be the only accessible site of disease [12]. Delay in performing marrow studies risks prolonging uncertainty and allowing the disease to progress unchecked.

The aggressive nature of IVBCL is well documented. Outcomes are uniformly poor if diagnosis is delayed, as emphasized in natural history studies [8]. The introduction of rituximab in combination with anthracycline-based regimens, particularly R-CHOP, has transformed the management of this disease and significantly improved survival compared with the pre-rituximab era [3,4]. Timely initiation of treatment is essential, however. In this patient, HLH-directed therapy provided only temporary improvement by suppressing inflammation, without addressing the underlying lymphoma. This highlights the need for early recognition so that definitive treatment can be initiated.

In summary, this case illustrates how IVBCL can mimic HLH and evade detection on PET-CT, only to be revealed through a bone marrow biopsy. Awareness of the Asian variant and its clinical profile, along with an understanding of the limitations of imaging, can help clinicians consider IVBCL sooner in patients with unexplained fever, cytopenias, and hepatosplenomegaly. Early suspicion and prompt biopsy are critical because effective treatment is available, but only if the disease is identified in time.

## Conclusions

IVBCL is a rare but potentially treatable cause of PUO and secondary HLH. Its distinctive intravascular growth pattern explains the absence of lymphadenopathy and the nonspecific findings on imaging, factors that frequently contribute to diagnostic delay. Clinicians should maintain a high index of suspicion for IVBCL in elderly patients presenting with unexplained fever, progressive cytopenias, hepatosplenomegaly, and hyperferritinemia once infectious and autoimmune causes have been excluded. Importantly, a negative PET-CT does not rule out the disease; bone marrow biopsy remains the most reliable diagnostic tool. Prompt recognition and initiation of therapy can significantly alter the otherwise poor natural history of this lymphoma.
